# Utilization outcomes of a cancer rehabilitation (CRNav) program: getting to the quadruple aim in cancer care

**DOI:** 10.1007/s00520-025-09388-8

**Published:** 2025-04-05

**Authors:** Shana E. Harrington, Nicole L. Stout, Ashley W. Perry, Mindi R. Manes, Meryl J. Alappattu, Kailyn Horn

**Affiliations:** 1 Department of Exercise Science, Physical Therapy Program, Arnold School of Public Health, University of South Carolina, 1300 Wheat Street, Blatt PE Center, 101H, Columbia, SC 29208 USA; 2 Department of Hematology/Oncology, School of Medicine, West Virginia University, Morgantown, WV USA; 3 Department of Health Policy, Management, and Leadership, School of Public Health, West Virginia University, Morgantown, WV USA; 4 Halifax Health|Brooks Rehabilitation, Daytona Beach, FL USA; 5 Present Address: Brooks Rehabilitation, Jacksonville, FL USA; 6 Department of Physical Therapy, University of Florida, Gainesville, FL USA; 7 Survivorship and Wellness, American Cancer Society, Atlanta, GA United States

**Keywords:** Chronic disease, Integrated delivery systems, Patient assessment/satisfaction, Patient outcomes/functional status/ADLs/IADLs, Rehabilitation services, Quadruple aim

## Abstract

**Background:**

A cancer rehabilitation navigation (CRNav) program is an evidence-based care delivery model that uses a rehabilitation professional in the navigation role to support oncology care delivery, provide functional screening for early identification of impairment, and coordinate care delivery services to optimize early rehabilitation. There is limited research showing how a CRNav impacts healthcare utilization. The objective of this study was to assess utilization data for a CRNav Program and demonstrate how the program influences the effectiveness of cancer care delivery and patient and provider satisfaction.

**Methods:**

Data was collected from the electronic health record of the Brooks Rehabilitation/Halifax systems at a community cancer center to assess program and service utilization over 3.2 years using a retrospective design.

**Results:**

Over 3.2 years, the CRNav program received 1585 referrals and screened 1447 (91.3%) patients. Of the 1447 screenings performed, 73.6% were recommended to receive outpatient rehabilitation (*n* = 1065). Among patients screened, breast cancer was the most common cancer diagnosis (47%) followed by head and neck cancers (14%). There were 638 total rehabilitation visits identified for patients who were seen for services within the health system, with physical therapy encounters accounting for the greatest number (*n* = 462). The most common reasons for receiving physical therapy services included lymphedema (27%), pain (25%), and limited range of motion (12%). Patients reported high satisfaction (≥ 95.4%) in the areas of how well rehabilitation met expectations and overall satisfaction with the rehabilitation experience.

**Conclusions:**

Using a CRNav in a community cancer center resulted in efficient care of patients with cancer, improved patient satisfaction and patient outcomes, and an enhanced clinician experience. This program provides a value-based approach to care supporting the quadruple aim and improving the identification and management of cancer-related functional morbidity.

**Supplementary Information:**

The online version contains supplementary material available at 10.1007/s00520-025-09388-8.

## Introduction

The number of people living with and beyond cancer in the United States continues to increase yearly due to early detection, treatment advances, and the growth and aging of the population [1] Today there are more than 18 million Americans with a history of cancer and the number of survivors is expected to increase to 26 million by 2040 [2]. While more individuals are living longer after cancer, they face a variety of adverse side effects that lead to functional morbidity and reduced quality of life [3–6]. There is a substantial cost burden associated with survivors’ morbidity attributed to medical care and medications [7], as well as chronic or persistent physical impairments [8]. Not only does this contribute to high levels of work disability among survivors, but out-of-pocket costs increasingly contribute to financial morbidity.

Many cancer-related functional morbidities are amenable to rehabilitation interventions during all phases of survivorship [9]. It is recommended that rehabilitation occur as early as possible in the cancer care continuum to facilitate early identification and management of impairments to reduce functional morbidity [10–15]. Despite this, only 9–12% of survivors are referred to rehabilitation [16, 17]. Emerging care delivery models for cancer survivorship and rehabilitation suggest that these supportive services be integrated with cancer care to achieve better treatment and health service outcomes [16, 18]. While evidence suggests there may be benefits to overall healthcare utilization and cost associated with early rehabilitative interventions, further investigation of these endpoints is needed [10, 19, 20].

A Cancer Rehabilitation Navigation (CRNav) program is an evidence-based care delivery model [21, 22] that uses a rehabilitation professional in the navigation role to support oncology care delivery, provide functional screening for early identification of impairment, and coordinate care delivery services to optimize early rehabilitation [21]. Using a prospective surveillance care delivery approach [10, 23], the navigator conducts a functional assessment at the time of cancer diagnosis and continues at intervals throughout cancer treatment to facilitate early intervention with rehabilitation services. This approach has the potential to improve patient experience, reduce provider burden in coordinating services, and enhance patient outcomes.

A CRNav program was implemented through a joint venture between Brooks Rehabilitation and Halifax Health in 2019. To date, the navigator has consulted on > 1100 patients for symptom and functional morbidity management. Methodically studying this successful program implementation is useful to inform future efforts that may seek to implement programs such as this into cancer care delivery [24]. The purpose of this manuscript is to share the utilization data from the CRNav program and demonstrate how the program can align to help healthcare systems and cancer centers achieve the Quadruple Aim [25–27]. While the implementation of a CRNav program has recently been studied to characterize the factors that contribute to successful implementation [22], to our knowledge, there is no published research describing the healthcare utilization outcomes of these programs.

## Methods

The CRNav program was developed out of a community need and not originally developed for research purposes. This work is part of a larger project that conducted a post-implementation analysis of a CRNav program in a community cancer center to describe implementation barriers and facilitators, outline the program workflow, and establish consensus on multilevel interventions to facilitate CRNav implementation. These results are published elsewhere [22]. Data was also collected from the electronic health records of the Halifax Health systems to assess program and service utilization. This project received Institutional Review Board Approval from the University of South Carolina (Pro00119001). Because this research presented no more than minimal risk to human subjects, due to data being de-identified when accessed, the study received an expedited review in accordance with the ethical standards set by the University of South Carolina’s Institutional Review Board.

### Care delivery model

**Fig. 1:**
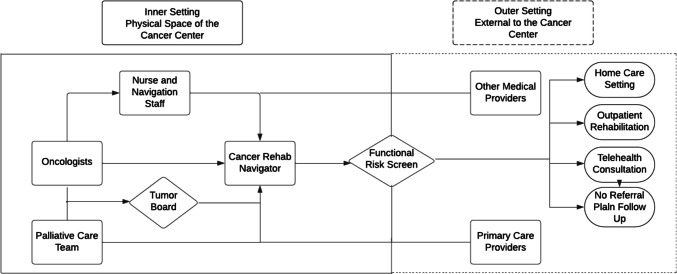
CRNav process workflow

A cancer rehabilitation navigator (CRNav) was co-located in the cancer center to perform functional screening on patients at the time of diagnosis, to consult on functional issues that arose through the duration of active treatment, and to coordinate care to manage functional morbidity using system and community resources. The CRNav process workflow is depicted in Fig. 1. In the joint venture with Brooks/Halifax Health, six rehabilitation clinics are located in the region. There are 17 PTs, four OTs, and three SLPs. Of these 26 rehabilitation providers, 20 (77%) have received advanced training in Oncology Rehabiltation. Other referral sources include registered dieticians, social work, financial services assistants, and disease (cancer) navigators for breast, gastrointestinal, head and neck cancer, and neurologic impairments. Program characteristics, sources of data, and extraction methods are described below.

### Cancer rehabilitation navigator

The CRNav is a doctor of physical therapy with 13 years of experience who completed post-professional training in oncology rehabilitation and an advanced residency program in orthopedic physical therapy. The CRNav has been the oncology program coordinator at Brooks Rehabilitation/Halifax Health since 2017 and has completed over 300 continuing education hours in oncology rehabilitation.

### Referral sources

The cancer center has three departments including Medical Oncology, Radiation Oncology, and Gynecologic Surgical Oncology. There are nine Medical Oncologists and three Advanced Practice Registered Nurses (APRNs), two Radiation Oncologists, one Gynecologic Oncology Surgeon, and one APRN. The patient care navigation team has five navigators in addition to the CRNav which are divided by specialty including breast, lung, gastrointestinal, survivorship, head and neck, and neurological cancers. In addition to the main campus, the Cancer Center for Hope has two other locations for medical oncology. The CRNav accepts referrals for consultation from any of the three locations within the Halifax Health Oncology service line. The main sources for referrals are the Medical Oncology, Radiation Oncology, Gynecologic Oncology, or their respective APRNs within the cancer center. Additional internal referral sources include radiation or medical oncology registered nurses, patient care navigators, dieticians, social workers, and radiation therapists. External referrals, which are those received outside of the Brooks Rehabilitation/Halifax Health System include general surgeons, breast surgeons, interventional radiologists, and/or primary care providers. Patients may also be referred to the CRNav from tertiary specialty centers in the Daytona Beach, Florida region.

### Referral procedures

Due to the high volume at the oncology center, it is not feasible for the CRNav to see all patients under the care of an oncologist. Referral pathways were created based on risk for impairment or functional decline (e.g., type of cancer, extent of surgery, stage of cancer) to drive appropriate patients to the CRNav for screening. The risk-stratified pathway was based on the clinical assessment by the navigator, using an internally developed screening tool (Supplemental Material). The tool is not yet validated; however, the accumulation of toxicity-inducing therapies alongside the clinical judgment of the navigator contributed to the risk assessment. The work on validating this tool is in process and is beyond the scope of this paper to present the tool here.

Further, any oncologist may refer patients directly when impairments are reported by the patient or identified on exam throughout the care continuum. Extensive education regarding functional morbidity and rehabilitation indications is provided to the oncology teams on an ongoing basis to facilitate referrals to the CRNav program.

### Screening procedures

Upon receipt of the referral, the patient is screened to determine if physical limitations are present or if a change in physical function is reported or identified by the provider during cancer treatment. Screenings are performed either in person or via telephone. Because screening by the navigator was meant to be quick, often using yes/no questions and identifying the need for further assessment, no specific outcomes were collected [28] (Supplemental Material). If screening revealed no deficits and the patient has a less extensive cancer treatment plan, they were classified as lower risk and provided with education on the sequelae of their specific cancer treatments, according to the American College of Sports Medicine (ACSM) movement guidelines [29], how to identify emerging impairments, and a home exercise program appropriate for the cancer type and patient preferences. If at any time a patient’s functional activities are negatively affected or prevent them from maintaining activity levels as identified by the patient, caregiver, or oncology team, they could be re-evaluated by the CRNav to determine the appropriateness of rehabilitation services [10].

### Sources of data

Data for this study were obtained from two sources: the CRNav patient database and hospital referral data for the CRNav program from October 1, 2019, to December 31, 2022. A secure, electronic patient database, using Microsoft Excel (2016), is maintained for all patients referred to the CRNav program. The database is a tracking tool for the CRNav to collect patient demographics, disease characteristics, and screening results and to characterize referrals made by the CRNav based on findings. Data was collected for each program year (2019–2022).

Electronic medical record patient-level data including patient age, gender, and variables related to the outpatient episode of care including total rehabilitation visits and the average length of the outpatient episode were extracted for all individuals in the CRNav database. Patient experience surveys are conducted by the Brooks Rehabilitation system to assess patient satisfaction using a percent global rating scale. This survey is administered via patient email after discharge from rehabilitation. The survey does not ask specific questions about the navigator and is included as Supplemental Material. These data were extracted from the EHR for each patient in the CRNav database.

Individual identifiers were used to merge the CRNav patient database with Halifax Health EHR and patient experience data.

### Analysis

The analysis was conducted based on three of the four domains of The Quadruple Aim Framework using a conceptual model. The quadruple aim suggests that improving the U.S. healthcare system involves the simultaneous pursuit of (1) improving the patient experience of care (including quality and satisfaction), (2) improving the health of populations, (3) reducing the per capita costs of healthcare, and (4) improving the work life of healthcare providers, including clinicians and staff [25, 30]. Results were analyzed to examine CRNav *Care Efficiency*, defined as the time from identification of rehabilitation need to the receipt of services, using the time from CRNav screen to rehabilitation evaluation. *Patient Experience*, based on the health system satisfaction data, and *Patient Outcomes* are defined using three variables of interest: (1) the total and average number of rehabilitation visits, (2) cancer diagnosis, and (3) rehabilitation diagnoses.

A screening rate (percentage of referrals screened) was calculated to quantify the number of referrals received and screened by the CRNav (screens/referrals = percentage), the frequency of screens recommended for outpatient rehabilitation, and the percentage of patients recommended for outpatient rehabilitation who were evaluated in the Halifax Health system (screens/services at Halifax Health = percentage). Percentages were also calculated for those who were referred to home health, hospice, skilled nursing, inpatient rehabilitation, and for those not medically safe for rehabilitation.

An episode of outpatient care was defined as the time between admission (date of first visit) and discharge (date of last visit). Patients may have received therapy from multiple disciplines during an episode of care.

## Results

### Care efficiency

Since the CRNav program’s inception, 1585 referrals were received, and 1447 (91.3%) patients were screened. Reasons for not being screened included the patient being unable to be contacted or the patient dying at the time of referral to screening. Of the 1447 screenings performed, 73.6% were recommended for outpatient rehabilitation (*n* = 1,065) and 5.2% (*n* = 75) were not medically safe for rehabilitation. Reasons a patient was deemed not medically safe included unstable disease that would require surgical intervention, metastases through the cortices, and medical instability. Data were incomplete for 3.9% of records (*n* = 18) which can be common with clinical data [31]. Six hundred and six patients screened by the CRNav received outpatient rehabilitation within the Brooks Rehabilitation System. Reasons why a patient who was recommended for rehabilitation did not seek it within the Brooks Rehabilitation System were not specifically documented. However, the CRNav did report several patients stating their insurance was not accepted in the system or they preferred to be a rehabilitation clinic closer to their home that was not a Brooks entity. Additional information on the health system referral, screens, and rehabilitation episodes of care can be viewed in Table 1.

**Table 1:** Health system referrals, screens, and rehabilitation data

Year	Referrals	Screens	Percentage of referrals screened	Screens recommended for outpatient rehabilitation	Screens recommended to other services	Not medically safe for rehabilitation	Received outpatient rehabilitation in Brooks System
2019^*^	89	80	89.9%	68 (85.0%)	Home health = 8 (10.0%) Hospice = 1 (< 1%) SNF = 1 (< 1%)	0	27 (33.8%)
**2020**	461	424	92.0%	303 (71.5%)	Home health = 44 (10.4%) Hospice = 3 (< 1%) SNF = 2 (< 1%) Inpatient = 4 (< 1%)	7 (1.7%)	169 (39.9%)
**2021**	531	480	90.4%	384 (80%)	Home health = 49 (10.2%) Hospice = 2 (< 1%) SNF = 1 (< 1%) Inpatient = 1 (< 1%)	38 (7.9%)	206 (42.9%)
**2022**^^^	504	463	91.9%	310 (70.0%)^^^	Home health = 40 (8.6%) Hospice = 7 (1.5%) SNF = 1 (< 1%) Inpatient = 4 (< 1%)	30 (6.5%)	204 (44.0%)
**Total**	1585	1447	91.3%	1,065 (73.6%)	Home health = 141 (9.7%) Hospice = 13 (< 1%) SNF = 5 (< 1%)	75 (5.2%)	606 (41.9%)

^*^CRNav program started in October

^^^Missing data

### Patient experience

Sixteen percent of those who received outpatient rehabilitation at Brooks responded to the patient experience survey. Patients reported high satisfaction (≥ 95.4%) in the areas of how well rehabilitation met expectations and overall satisfaction with the rehabilitation experience. Ninety-six percent of patients reported they would recommend Brooks Rehabilitation and 96% reported they would return if they needed rehabilitation again (Table 2).

**Table 2:** Outpatient rehabilitation descriptive data, *n* = 606

Characteristics	Value
Female	431(71%)
Male	175 (29%)
Age (years)	66.8(11.7)
Average time from screen to rehabilitation evaluation (days)	15.7(12.6)
**Total number of rehabilitation visits** (*n* = 638)	
PT	462 (72%)
OT	103 (16%)
SLP	73 (11%)
**Average number of rehabilitation visits**	
PT	8.38(10.9)
OT	1.44(4.71)
SLP	1.00(3.88)
**Patient experience (satisfaction)**	
Met expectations	95.4%
Overall satisfaction	95.7%
Would recommend	96.0%
Would return	96.0%

### Patient Outcomes

**Fig. 2:**
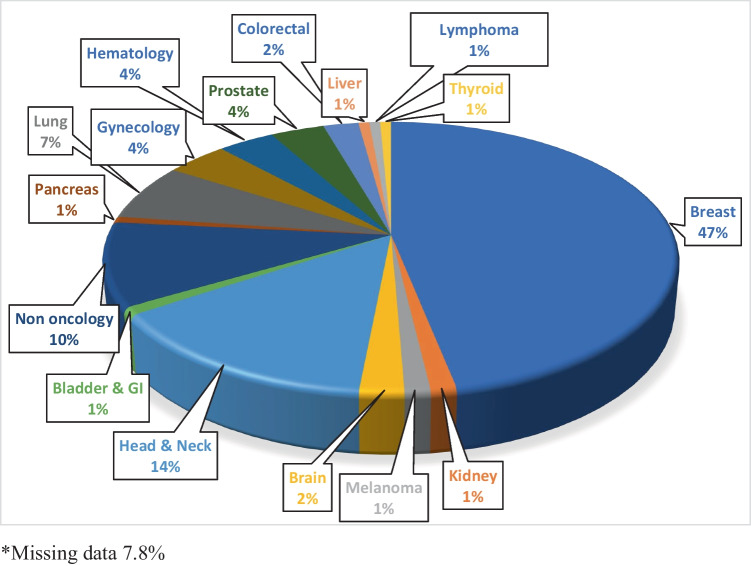
Type of cancer diagnoses

**Fig. 3:**
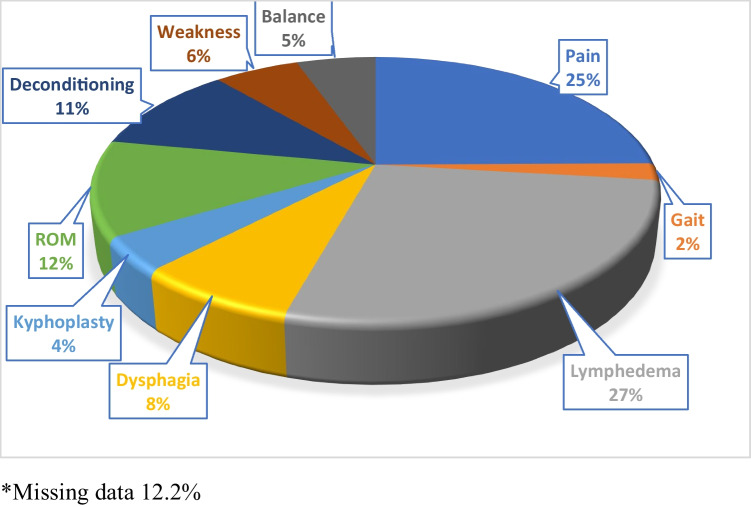
Rehabilitation diagnoses

Patient characteristics, including gender, age, the average time from screening to rehabilitation (days), and the total number, and the average number of rehabilitation (PT, OT, and SLP) visits, for those who received outpatient rehabilitation in the Brooks System, can be viewed in Table 2. There were 638 total rehabilitation visits during the period of study, with physical therapy accounting for the greatest number (*n* = 462). The average number of rehabilitation visits was 8.38 (10.9), 1.44 (4.71), and 1.00 (3.88) for physical therapy, occupational therapy, and speech-language pathology respectively, with 33 (5.5%) receiving multidisciplinary rehabilitation. Figure 2 shows breast cancer was the most common cancer diagnosis (47%) followed by head and neck cancer (14%). It is important to note that approximately eight percent of the patients had no documented data on the type of cancer diagnosis and 10 percent of those screened by the CRNav did not have a cancer diagnosis. This is because the setting the CRNav works in allows for screening of patients with other diagnoses including transplant, benign hematology, and those receiving kyphoplasty. The most common reasons for rehabilitation referral, which are deciphered by coders to document the appropriate ICD-10, included lymphedema (27%), pain (25%), and limited range of motion (12%) as seen in Fig. 3. Approximately 12% of patients did not have a documented rehabilitation diagnosis.

## Discussion

Cancer care delivery is exceedingly complex and inherent in all cancer treatments is the risk for functional morbidity. As functional morbidity becomes more prevalent in a growing number of survivors, it is critical to implement evidence-based interventions that can improve long-term survivorship as well as improve care coordination to facilitate better functional outcomes. Cancer rehabilitation navigation combines the roles of patient navigators and cancer rehabilitation specialists to facilitate functional screening and care coordination for evidence-based rehabilitation interventions [21]. While patient navigation services have demonstrable cost and clinical effectiveness [32, 33], understanding how expanding navigation to include functional assessment can impact care efficiency, quality, and patient and provider experience, as suggested by the Quadruple Aim, is needed to support wider adoption of integrated cancer rehabilitation care delivery models. We used the Quadruple Aim as a conceptual model to quantify the benefits of a CRNav program in the domains of care efficiency, patient experience, and patient outcomes, and, based on previous findings from this project, provide insight into how this program influences clinician experience.

### Care efficiency

It is well-documented that cancer places a large financial burden on society [34, 35]. Identifying cancer-related functional impairments early, using a prospective surveillance approach [10], throughout the cancer care continuum may reduce the severity and incidence of future disability [34, 36]. Embedding a CRNav in the Brooks Rehabilitation/Halifax Health System allowed our patients to receive a rehabilitation evaluation within 16 days (= / − 12.6) of screening positive for an emerging functional impairment. This is important to long-term survivorship because early cancer rehabilitation can improve physical function, reduce psychological distress, and promote a return to work, positively impacting both direct and indirect healthcare costs [10, 34, 37–41]. As the CRNav model is new, there are few published reports describing outcomes of efficiency, therefore making comparisons difficult. It is important to point out that 25% of our patients were seen for a rehabilitation evaluation within seven days of positive screening with the CRNav.

It has been documented that impairments caused by a cancer diagnosis and treatments can increase the utilization of rehabilitation [8, 42]. This often happens in the traditional model of care when rehabilitation utilization is typically reactive. In these circumstances, patients often present with multiple impairments, many at higher levels of severity, which have impacted their function and quality of life for some time [10, 19, 43]. While it is challenging to estimate the direct and indirect healthcare costs of impairments related to cancer, emerging studies suggest cancer rehabilitation, especially early rehabilitation can be cost-effective [10, 19, 34]. In a systematic review conducted by Mewes et al. [19] on the cost-effectiveness of cancer rehabilitation, all four studies reviewed showed favorable cost-effectiveness ratios, although the authors reported a wide range of rehabilitation interventions implemented. Stout et al. [10] reported significant cost-savings when managing early-stage breast cancer-related lymphedema per patient per year when using a prospective surveillance model. Additionally, because we do not have a comparison group of individuals who did not receive early rehabilitation, we are unable to directly infer the overall cost savings in using a CRNav. Future research should include a control group to perform a cost-effectiveness analysis.

### Patient experience

An influential dimension of healthcare quality is the level to which healthcare services meet the needs of those who use them, also known as the patient experience [30, 44]. A recent study by Wood et al. [45] retrospectively examined medical record data of 383 patients with cancer who received outpatient rehabilitation to better understand patient experience using the Net Promoter Survey® [46]. Because the survey used by Brooks and the Net Promoter Survey® asks “How likely are you to recommend this facility to family/friends?,” an appropriate comparison. This study found a 91.4% satisfaction score, slightly lower than our report of 96%. Unfortunately, Wood et al. did not report a standard deviation making effect size calculations impossible for further comparison. Additionally, the authors did not report the time since diagnosis making comparisons to our study difficult. Nonetheless, our reported overall patient satisfaction score suggests a positive patient experience with receiving rehabilitation services.

### Patient Outcomes

The average number of visits for rehabilitation in our study was considerably lower than in previous reports. Wood et al. examined the number of patient visits but aggregated occupational and physical therapy visits, reporting an average of 14.23 (12.37) visits attended. Our reported number of visits of 8.38 for PT and 1.44 for OT may reflect that our patients needed a lower number of visits due to early identification, however. Wood et al. [45] did not report the time since cancer diagnosis making comparisons to the average number of visits in our study difficult. Additionally, the study conducted by Wood et al. reported data during “appointments in 2019”, whereas our study reported data during a longer period (October 2019–December 2022). A retrospective chart review over 2 years conducted on 418 adults with cancer who received physical therapy reported an average of 8.5 (11) sessions [47], which is similar to what was found in our study of 8.4 (11) physical therapy visits. The study by Alappattu et al. [47] reported adults with genitourinary cancer were the most common cancer type in their study at 40.4%, whereas our study had small numbers of genitourinary cancers (5%). Since other rehabilitation disciplines were not reported as well as the time since cancer diagnosis, additional comparisons are difficult to ascertain.

Cancer and its treatments are known to cause a variety of impairments that impact physical function [48]. The top three primary impairments that were identified requiring rehabilitation referrals in our study were (1) lymphedema (27%), pain (25%), and limited range of motion (12%). We are aware of one study that described clinical characteristics in 418 adults with cancer referred for outpatient physical therapy as previously mentioned, although 18% lacked impairment data [47]. It is important to note that more than one impairment was reported, and impairments were described as a total percentage of individuals with the impairment, whereas our study reported only the primary impairment. The top three impairments reported in the study by Alappattu et al. were strength (83.6%), soft tissue (71.3%), and limited range of motion (51.3%) [47]. This information in aggregate suggests that impairments may vary based on the type of cancer and the body systems impacted by treatment, therefore successful screening approaches should consider functional impairments broadly, across multiple body systems known to be impacted by cancer treatments [5].

### Clinician experience

Improved clinician experience focuses on the care of the provider in optimizing the performance of the healthcare experience [49]. This aim addresses the vital role clinicians have in the successful adoption of care delivery interventions as well as the challenges of clinician burnout [30, 50].

Previously published qualitative findings from this project characterize the influence and positive impact of the CRNav and the CRNav program on cancer care providers including medical and radiation oncologists and oncology nurses and navigators [22]. Facilitating strategies, that contributed to the successful implementation of the CRNav program included *supporting oncology clinicians*, *creating multi-disciplinary cancer teams*, and *educating rehabilitation clinicians*, and *program staff* [22]. These facilitators of implementation improved clinician acceptability of the CRNav program as well as provider satisfaction as evidenced by qualitative findings [22].

The issue of burnout among oncology healthcare professionals is receiving increasing attention. While clinic volume and condition complexity in cancer are identified as primary factors contributing to burnout, additional administrative tasks, and patient care tasks not aligned with the providers’ workload are variables that may also contribute to burnout [51, 52]. We posit that CRNav may help to alleviate the workload burden of oncology providers by taking responsibility for coordinating care to manage the functional impairments that many patients face. This was highlighted in recent qualitative findings associated with this project, physician providers felt relieved that the CRNav could not only resolve issues related to poor function but manage care coordination in that regard. Further, team-based care delivery approaches also enhance the clinician experience [52], suggesting that a CRNav, functioning as part of the care team could be beneficial.

A recent study by Brennan et al. explored the experiences of physical therapists in cancer care practice in Ireland [53]. Findings suggest that having good support from their health system to undertake continuing professional development leads to higher satisfaction [53]. Additionally, support from hospital management, oncology, and service teams was important in the development of cancer services for physical therapy [53]. This study, along with our recently published study [22] are notable examples of how the CRNav improves clinician engagement and support in cancer care delivery models.

## Limitations

All CRNav screenings were conducted by a single person and took place in one health system (Halifax Health), restricting generalizability to other health systems. There was missing data throughout the 27-month data period and missing variables such as date of cancer diagnosis, stage of cancer, and an objective physical function measure were not reported. This CRNav did not assess baseline function, before cancer treatment as recommended in the Prospective Surveillance Model by Stout and colleagues [43]. Information about the utilization of other healthcare services is beyond the scope of this study, although important for future research. Future research should work to highlight the importance of researcher and clinical provider relationships to help minimize missing data and provide the optimal variables to best drive decision-making. The quadruple aim has evolved since the COVID-19 pandemic and a fifth aim has been proposed which incorporates health equity that should be addressed in future CRNav health services research [54].

## Conclusion

In this study, which is ongoing, we used the Quadruple Aim as a conceptual model to synthesize the healthcare service utilization outcomes of a CRNav program to screen and manage patient physical function. We found that the CRNav integration in cancer care resulted in efficient identification and management of functional impairment, promoted high patient satisfaction and patient outcomes, and enhanced clinician experience, supporting the quadruple aim. Future research is needed to explore the impact of a CRNav program in a prospective, multi-site study and to incorporate measures of health equity.

## Supplementary Information

Below is the link to the electronic supplementary material.

Supplementary file1 (DOC 99 KB)

Supplementary file2 (PDF 124 KB)

## Data Availability

Data specific to this study can be requested from the lead author.
