# Supplementary material for: Utilization outcomes of a cancer rehabilitation (CRNav) program: getting to the quadruple aim in cancer care
Source: Support Care Cancer. 2025 Apr 5;33(4):357. doi: 10.1007/s00520-025-09388-8 (PMC11972230; doi:10.1007/s00520-025-09388-8)
Supplement: Supplementary file 1 — Supplementary file1 (DOC 99 KB) [file 520_2025_9388_MOESM1_ESM.doc]

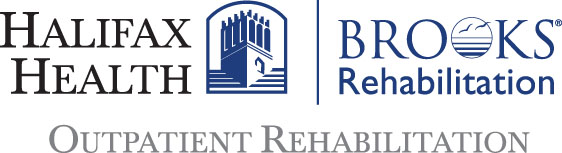


Patient Name : , Date:

Referring MD:

Dx:

PT Dx:

Medical Hx:

**Chemo agent**  Date:

Cardio Toxic:

1. Do you feel like weakness is effecting your ability to complete daily activities? Yes No
2. Do you feel too tired to do the things you would like? Yes No

Neuro Toxic:

1. Have you noticed a decline in your balance, do you feel unsteady on your feet?

Yes No

1. Any numbness/tingling in the extremities that effects your daily function or fine motor skills? Yes No
2. Any new onset of vertigo or dizziness? Yes No

**Radiation**: Date:

(screen for swallow or cardiac deficits if indicated)

**Surgery**: Date:

Lymph Node Resection : ALND SLND Not sure

1) Have you noticed heaviness or increase in swelling in the extremity? Yes No

2) Do you experience any tightness or decreased ROM around the surgical area? Yes No

**Functional Screen Questions**

1. Do you have any difficulty moving your head for talking to others, or looking side to side for driving? Yes No
2. Are you having any difficulty swallowing?

Yes No

1. Do you have any difficulty reaching overhead, into cabinets, reaching behind the back or carrying heavy items?

Yes No

1. Are you having any difficulty getting dressed, bathing, or taking care of yourself?

Yes No

1. Are you having difficulty completing moderate activity around your house such as, carrying groceries, lifting a gallon of milk, doing laundry or preparing meals?

Yes No

1. Are you having any pain that is limiting your function? (ie Shoulder , Neck, Jaw, AIs)

Yes No

1. Do you feel unsteady when standing or walking, are you able to walk on grass, down the driveway and negotiate a curb safely?

Yes No

1. Does Fatigue or weakness interfere with your ability to complete you daily activities?

Yes No

1. Have you noticed an increase in effort required with getting up and down from chairs, sofas, or toilets?

Yes No

1. 1) Have you had any falls /near falls in the past 3 months or do you feel the need to reach out for furniture or walls for stability?

Yes No

1. Do you feel limited in your ability to ambulate in the community for prolonged distances such as shopping or doctors appointments?

Yes No

(Answering Yes to any of these questions warrants referral to therapy)

**Fatigue Scale**

On a scale of 0 -10 how would you rate your level of fatigue in the past week

1 2 3 4 5 6 7 8 9 10

(Anything over 4 warrants referral)

**Summary:**

**Eval Date:**
