# Supplementary material for: Utilization outcomes of a cancer rehabilitation (CRNav) program: getting to the quadruple aim in cancer care
Source: Support Care Cancer. 2025 Apr 5;33(4):357. doi: 10.1007/s00520-025-09388-8 (PMC11972230; doi:10.1007/s00520-025-09388-8)
Supplement: Supplementary file 2 — Supplementary file2 (PDF 124 KB) [file 520_2025_9388_MOESM2_ESM.pdf]

BROOKS Rehabilitation

Brooks Rehabilitation strives to deliver valuable services to you, our customer. Thank you for taking time to complete this survey so that we can provide very good service and by using your feedback, improve for the future. Please return it in the self-addressed stamped envelope provided. For more information about Brooks, visit our website www.BrooksRehab.org.

Follow us: Twitter.com/BrooksRehab - YouTube.com/BrooksRehab - Facebook.com/BrooksRehab

1. **Please rate your overall therapy experience based on the following statements, *(please put an X in the box that best describes your experience or if not applicable please mark N/A)* :**

	Very Good	Good	Fair	Poor	Very Poor	N/A
How well therapy met my expectations.						
The value in my overall therapy experience was…						
The likelihood that I would recommend Brooks Rehabilitation is…						
The likelihood that I would return to Brooks if I needed therapy again is…						

2. **Please rate the following statements about the Physical Therapists or Therapist Assistants who treated you, *(please put an X in the box that best describes your experience or if not applicable please mark N/A)* :**

	Very Good	Good	Fair	Poor	Very Poor	N/A
The explanation of my evaluation was…						
The explanation of my plan for treatment was…						
The explanation of my progress in therapy was…						
The updates to my treatment program were…						
The education provided to me was…						
The therapist's concern for my privacy was…						

3. **Please rate the following statements about the Occupational Therapists or Therapist Assistants who treated you, *(please put an X in the box that best describes your experience or if not applicable please mark N/A*) :**

	Very Good	Good	Fair	Poor	Very Poor	N/A
The explanation of my evaluation was…						
The explanation of my plan for treatment was…						
The explanation of my progress in therapy was…						
The updates to my treatment program were…						
The education provided to me was…						
The therapist's concern for my privacy was…						

4. **Please rate the following statements about the Speech/Language Therapists who treated you, *(please put an X in the box that best describes your experience or if not applicable please mark N/A)* :**

	Very Good	Good	Fair	Poor	Very Poor	N/A
The explanation of my evaluation was…						
The explanation of my plan for treatment was…						
The explanation of my progress in therapy was…						
The updates to my treatment program were…						
The education provided to me was…						
The therapist's concern for my privacy was…						

5. **Facility *(please put an X in the box that best describes your experience)* :**

	Very Good	Good	Fair	Poor	Very Poor	N/A
Cleanliness of the waiting area was…						
Cleanliness of the treatment areas were…						

6. **Patient Registration and Scheduling: Please rate the following statements about the Front Desk Staff who assisted you, *(please put an X in the box that best describes your experience or if not applicable please mark N/A)*:**

	Very Good	Good	Fair	Poor	Very Poor	N/A
The professionalism of the Front Desk staff was…						
The helpfulness of the Front Desk staff was…						
The explanations of my benefits were…						
The assistance offered to me by the Front Desk staff during my registration was…						
The thoroughness in which my questions were answered by the Front Desk staff was…						
The scheduling of my appointments was…						

7. **Why did you choose our facility? Please check all that apply:**

☐ My choice
☐ Another Therapist
☐ Physician
☐ Friend
☐ Former Patient
☐ Internet
☐ Insurance Company
☐ Other

Other (please specify): ____

8. **Please tell us why you came to Therapy:**

☐ Joint or muscle pain (back, neck, knee, shoulder, hand, ankle and/or foot)
☐ Surgery in the last 3 months
☐ Stroke (CVA)
☐ Spinal Cord Injury
☐ Brain Injury
☐ Balance/Dizziness
☐ Sports Injury
☐ Therapy for my child
☐ Pelvic Floor disorders
☐ Pregnancy related pain
☐ Other Neurological conditions (Multiple Sclerosis, Parkinson's, ALS, etc.)

☐ Other (please specify): ____

9. **Please read the following statements and choose the response that most closely matches your opinion. (1 Strongly Disagree = 1, Disagree = 2, Agree = 3, Strongly Agree = 4)**

	1 Strongly Disagree	2 Disagree	3 Agree	4 Strongly Agree
During outpatient therapy, the staff took my preferences or those of my family/caregiver into account when deciding what my healthcare needs would be when I finished therapy.				
When I finished outpatient therapy, I had a good understanding of the things I was responsible for in managing my health.				

10. **Gender**

☐ Male ☐ Female

11. **Patient's Age**

☐ 0-5 years old
☐ 6-13 years old
☐ 14-21 years old
☐ 21-30 years old
☐ 31-40 years old
☐ 41-50 years old
☐ 51-60 years old
☐ 61-70 years old
☐ 71-80 years old
☐ 81-90 years old
☐ 90 years old and above

12. **Please enter any comments that you would like to make about your therapy experience.**

____

____

____

What would have made your experience more valuable?

____

____

NAME (optional)

____
